# P-269. From Missed Opportunities to Early Detection: A 10-Year Evaluation of HIV Testing Practices

**DOI:** 10.1093/ofid/ofaf695.490

**Published:** 2026-01-11

**Authors:** Andrea Pallotta, Francisco Marco Canosa, Caroline Feigert

**Affiliations:** Cleveland Clinic, Cleveland, Ohio; Cleveland Clinic Foundation, Cleveland, Ohio; Cleveland Clinic Foundation, Cleveland, Ohio

## Abstract

**Background:**

Human immunodeficiency virus (HIV) testing rates in healthcare settings vary, leading to potential missed diagnosis, advanced HIV, and continued risk of HIV transmission. Preliminary internal data of newly diagnosed patients from 2014-2019 revealed missed opportunities for previous HIV testing, outpatient diagnoses, and a high percentage of late-stage diagnoses. In 2020, a multidisciplinary, emergency department quality improvement initiative updated order sets to automatically include opt-out HIV testing when diagnosing sexually transmitted infections as well as an automatic forwarding of new HIV diagnoses to the Infectious Disease department. The current study describes demographics, risk factors, history of HIV testing, HIV stage and outcomes for new HIV diagnoses from 2020-2023 after intervention implementation.

Characteristics and outcomes of newly diagnosed HIV patients

**Figure:**
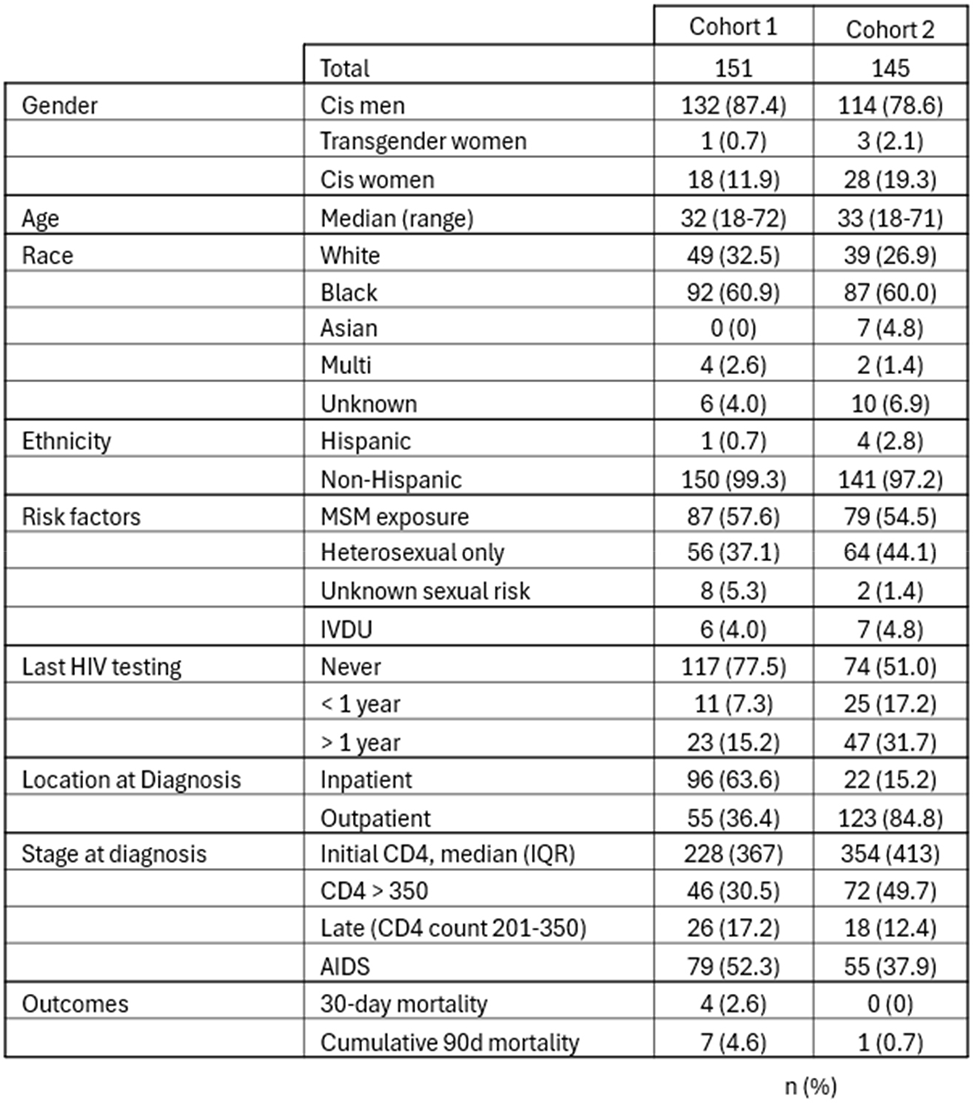

**Figure:**
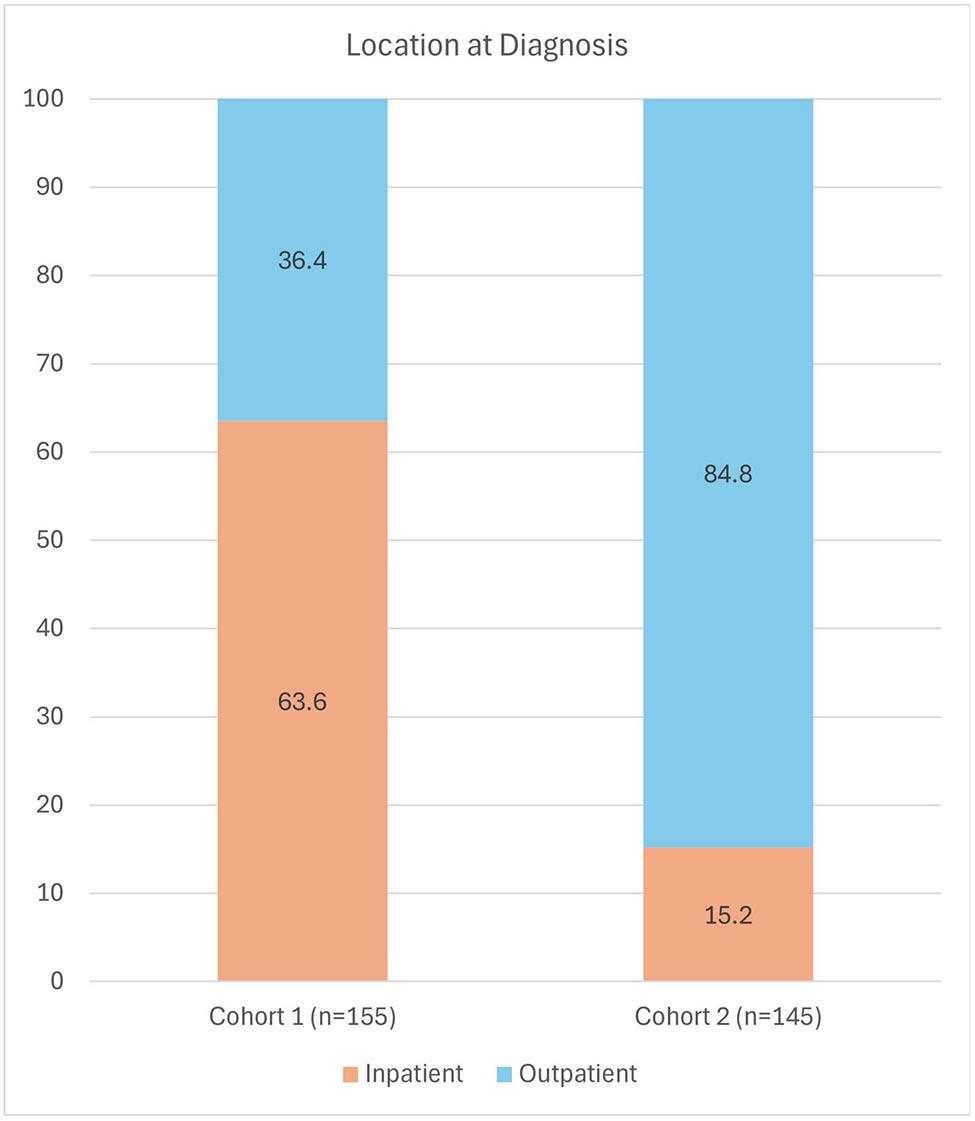

**Methods:**

Retrospective chart review of newly diagnosed HIV infection from 2014-2019 (unpublished data, “cohort 1”) and 2020-2023 (“cohort 2”). Data on demographics, acquisition risk, previous testing, diagnosis setting, CD4 count, viral load and 30-day mortality were collected. Previous HIV testing was determined from available lab results. Late diagnoses included patients with CD4 counts between 200 and 350. AIDS diagnosis was defined as CD4 counts less than 200 cells/uL, CD4% less than 15% and/or AIDS defining illness. Summary statistics were utilized.

**Results:**

New HIV diagnoses totaled 151 and 145 in cohorts 1 and 2, respectively. Both groups were male (87.4% and 78.6%), Black (60.9% and 60.0%) with transmission risk factor men who have sex with men (57.6% and 54.5%). Outpatient HIV diagnoses dramatically increased (36.4% vs 84.8%). Late presentation rates decreased (17.2% vs 12.4%). Overall AIDS diagnosis decreased (52.3% vs 37.9%), with median CD4 count at diagnosis increasing from 228 to 354. Thirty-day mortality rates in newly diagnosed patients with AIDS decreased between cohorts (2.6% vs 0%).

**Conclusion:**

The multidisciplinary intervention in 2020 increased outpatient screening for HIV, leading to fewer diagnoses at advanced stages.

**Disclosures:**

All Authors: No reported disclosures

